# mDARAL: A Multi-Radio Version for the DARAL Routing Algorithm

**DOI:** 10.3390/s17020324

**Published:** 2017-02-09

**Authors:** Francisco José Estévez, José María Castillo-Secilla, Jesús González, Joaquín Olivares, Peter Glösekötter

**Affiliations:** 1 Department of Electrical Engineering and Computer Science, University of Applied Sciences of Münster, Stegerwaldstr. 39, D-48565 Steinfurt, Germany; gloesek@ieee.org; 2 Department of Computer Architecture and Technology, University of Granada, Periodista Daniel Saucedo Aranda, S/N, 18071 Granada, Spain; jesusgonzalez@ugr.es; 3 Department of Computer Technology, University of Alicante, Carretera San Vicente del Raspeig, S/N, 03690 Alicante, Spain; jmcastillo@dtic.ua.es; 4 Department of Computer Architecture and Technology, University of Cordoba, Campus de Rabanales, 14001 Cordoba, Spain; olivares@uco.es

**Keywords:** multi-radio, network routing algorithm, WSN, DARAL, smart city, IEEE 802.15.4

## Abstract

Smart Cities are called to change the daily life of human beings. This concept permits improving the efficiency of our cities in several areas such as the use of water, energy consumption, waste treatment, and mobility both for people as well as vehicles throughout the city. This represents an interconnected scenario in which thousands of embedded devices need to work in a collaborative way both for sensing and modifying the environment properly. Under this scenario, the majority of devices will use wireless protocols for communicating among them, representing a challenge for optimizing the use of the electromagnetic spectrum. When the density of deployed nodes increases, the competition for using the physical medium becomes harder and, in consequence, traffic collisions will be higher, affecting data-rates in the communication process. This work presents *mDARAL*, a multi-radio routing algorithm based on the Dynamic and Adaptive Radio Algorithm (*DARAL*), which has the capability of isolating groups of nodes into sub-networks. The nodes of each sub-network will communicate among them using a dedicated radio frequency, thus isolating the use of the radio channel to a reduced number of nodes. Each sub-network will have a master node with two physical radios, one for communicating with its neighbours and the other for being the contact point among its group and other sub-networks. The communication among sub-networks is done through master nodes in a dedicated radio frequency. The algorithm works to maximize the overall performance of the network through the distribution of the traffic messages into unoccupied frequencies. The obtained results show that *mDARAL* achieves great improvement in terms of the number of control messages necessary to connect a node to the network, convergence time and energy consumption during the connection phase compared to *DARAL*.

## 1. Introduction

The rapid growth of Smart Cities among academics and public organizations is leading to numerous research projects [1,2,3]. Its rise involves the development of embedded devices, powering techniques and communication technologies. This work focuses on the challenge that communication technologies represent. This challenge has been intensively studied, as it is a large area of knowledge, which covers every aspect of the communication itself, such as traffic, collision reduction, data rates increase, number of nodes, compatibility, convergence time or even energy used by the different network protocols. Previous works of the authors focused on some of these aspects like the convergence time, energy used and control messages used by network protocols, proposing a new one, Dynamic and Adaptive Radio Protocol (DARP) [4,5], focused on the optimization of these parameters during the forming phase of the network.

The analysis of Smart City projects [6,7] and the increment in terms of number of nodes and density present in networks [8,9,10,11] have led to new challenges. The number of collisions and the saturation of the channels are problems that need new solutions and proposals. The study of multi-channel or even multi-radio approaches is a major trend today [12] for solving those issues. In [13], Bahl et al. demonstrate that a multi-radio system can provide high data rates with low energy consumption. Kusy et al. [14] analyzed how network reliability can be significantly improved with dual radio communication without having a significant impact on energy consumption. Following these works, the development of a new version of *DARP* with multi-radio support is a logical development in order to face the above-mentioned new challenges.

*mDARAL* proposes a multi-radio routing algorithm based on the Dynamic and Adaptive Radio Algorithm (*DARAL*). It uses the main functionality of *DARAL* [5], adding the capability of selecting the communication frequency for each sub-network, regarding the use of each channel. It intends to maximize the performance through the distribution of the traffic into unoccupied frequencies. Therefore, the algorithm offers a better load distribution, minimizing the overall collisions. Since *DARAL* organized the nodes in virtual sub-networks, each sub-network will adapt its frequency independently. *mDARAL* is designed to be used in multi-radio devices, using one radio only for relevant communications on a fixed channel. In order to reduce the power consumption, the use of the radios is optimized, disabling inactive radios in nodes without routing requirements.

The rest of the paper is organized as follows: Section 2 provides an overview of different previous works in the area of multi-radio based routing algorithms techniques for Wireless Sensor Networks (WSN). Section 3 describes *mDARAL*, the proposed algorithm designed for a better use of the available channels, increasing the delivery ratio in the network. The simulation procedure and the obtained results are shown in Section 4 and Section 5. Section 6 presents the conclusions of this paper.

## 2. Routing Algorithms and Multi-Radio Techniques for WSN

Smart City and Internet of Things (IoT) projects require the use routing algorithms to create the network topology. Several routing methods are used in these scenarios, like multicast, mesh or graphed based, clustering algorithms focused on improving the QoS, the energy consumption, the traffic reduction, the network lifetime, or the network adaptability. Our proposal, DARAL, falls within the clustering-based routing algorithms. Despite the fact that these algorithms are mainly hierarchical cluster-tree algorithms, there also exist interesting alternatives like the one proposed by Liu et al. in [15], Saravanan et al. in [16] or the one proposed by Wang et al. in [17].

It is usual to find two different roles for the nodes of a cluster-based network, a central node or cluster-head and another node, usually called the end-node. Clusters are formed by at least one cluster-head and several end-nodes. In the pure cluster tree algorithms, the cluster-head receives the traffic from all of the end-nodes, and sends it to the proper destination. Furthermore, it is also responsible for the communications between other cluster-heads from clusters with lower/higher hierarchy level. However, clustering mesh-like algorithms allow end-node to end-node communication, reducing the bottle neck in cluster-heads [18]. Therefore, the role selection mechanism is a fundamental aspect of these algorithms. There exist different cluster-head selection mechanisms that cover different approaches depending on different parameters such as location, Link Quality Indicator [19] (LQI) or residual energy [20]. In addition to the diversity of scenarios, there are also a wide number of different approaches to hierarchical cluster-based routing algorithms. Every routing algorithm intends to improve a particular group of parameters related to a specific scenario.

However, Machado et al. discussed in their article the dynamical nature of a Smart City or IoT scenario [21]. Therefore, routing solutions for those applications should take into account different traffic patterns such as one-to-many, many-to-one and many-to-many. Gkelias et al. [22] proposed a many-to-many routing algorithm which uses the multi-user gain capability of Multiple-Input Multiple-Output (MIMO) by controlling the number of ongoing transmissions in order to increase the end-to-end throughput and, thus, to guarantee the required QoS constraints. These concepts have led most of the proposals for Smart Cities or IoT applications to use two well-known routing algorithms: an Ad-Hoc On Demand Vector (AODV) and IPv6 Routing Protocol for Low-Power and Lossy Networks (RPL), due to their appropriate performance in these scenarios. Recent hierarchical routing algorithms such as *DARAL* [5] have proved themselves as interesting alternatives to the commonly used protocols.

AODV is an on-demand routing algorithm that discovers routes according to the network necessities. It uses request and reply messages to find the shortest path between the sender and the receiver. The network connections create a mesh structure, where all the nodes present the same hierarchy level. On the other hand, RPL routes are formed around a central node, which acts as a network root or network coordinator. This node broadcasts messages with its information in order to expand the network range as well as to keep the nodes connected. However, neither of these two protocols is ideally designed to be used in a Smart City scenario. AODV does not optimize the traffic overhead (the control messages sent to form the routes), obtaining poor energy consumption results, and RPL does not present an efficient consumption either, as it was discussed in [23]. Consequently, *DARAL* intended to solve the lack of these protocols.

*DARAL* can be described as a centralized non-beaconing routing algorithm based on dynamic clustering, which is achieved using the sub-network concept. A node must be defined as the network root or coordinator. Its neighbour nodes can connect to it using different roles, either as end nodes (ENs) or as virtual coordinators (VCs). Each VC is responsible for creating and maintaining a sub-network that is identified with a virtual identification (vID) as was described in [24]. A node will be treated as an EN when the link quality between it and its coordinator is higher than a certain threshold. If the quality is worse than this value, the node acquires the role of virtual coordinator, which will try to expand the network, creating a sub-network around it. Each VC can be connected to new ENs or VCs, producing a multi-level hierarchy of sub-networks. Therefore, the network is similar to a tree topology, where VCs are the element of a branch and ENs act as leaves in the tree. The dynamic role selection process (DSRP) is the most important aspect of the network formation. The appropriate number of VCs will depend on the scenario, so the threshold level is adaptable to each deployment.

However, neither AODV, nor RPL, nor *DARAL* are fully designed to be used under a high-density environment, like Smart Cities or even the IoT. The high spectrum occupancy requires new techniques in order to provide adaptability to the network environment [25,26]. As it was described in [27], new radio developments are based on multi-radio systems, where a radio senses its electromagnetic environment and can dynamically adjust some of its parameters to improve the communication, which leads to a dynamic environment where the routing process acquires a great complexity. The nodes need to be aware not only of their neighbouring initial conditions but also of their current status. New routing protocols need to be developed in order to provide this functionality.

As it has been previously described, the actual routing algorithms have some drawbacks in terms of efficient convergence time, optimized energy consumption and minimal control overhead during the network set-up phase. Therefore, we propose an improvement of the *DARAL* routing algorithm which takes advantage of multi-radio and multi-channel techniques selection in order to maximize the throughput of the network, maintaining or even improving the convergence time, energy consumption and control overhead during the set-up phase of the network.

## 3. Multi-Radio Dynamic and Adaptive Routing Algorithm (mDARAL)

This work focuses on expanding the features of *DARP*, adapting the *DARAL* algorithm to make it usable within a multi-radio and multi-channel environment. Based on the analysis of *DARP*, it has been observed that the clustering and the use of virtual sub-networks is suited very well to a multi-radio environment. Moreover, the development of *DARP* is focused on Smart Cities, where the density and number of nodes tend to increase, thus the multi-radio scheme seems an interesting option for future developments.

First of all, it has been intended to keep the functionality of *DARAL* (dynamical clustering based on the link quality between nodes), but including the required mechanisms to make possible handling a second radio. Moreover, other interesting features from *DARAL* are maintained, like the sub-network structure, the parallel execution on each sub-network and the Dynamical Role Selection Process (*DRSP*) algorithm used in the original *DARP* protocol.

Based on *DARAL*, we have carried out a deep analysis of the multi-radio scenarios, concluding that some of the basic features such as the state machine or the link quality indicator (LQI) based route selection algorithm offer a promising base for the development of a native multi-radio network protocol.

**Figure 1:**
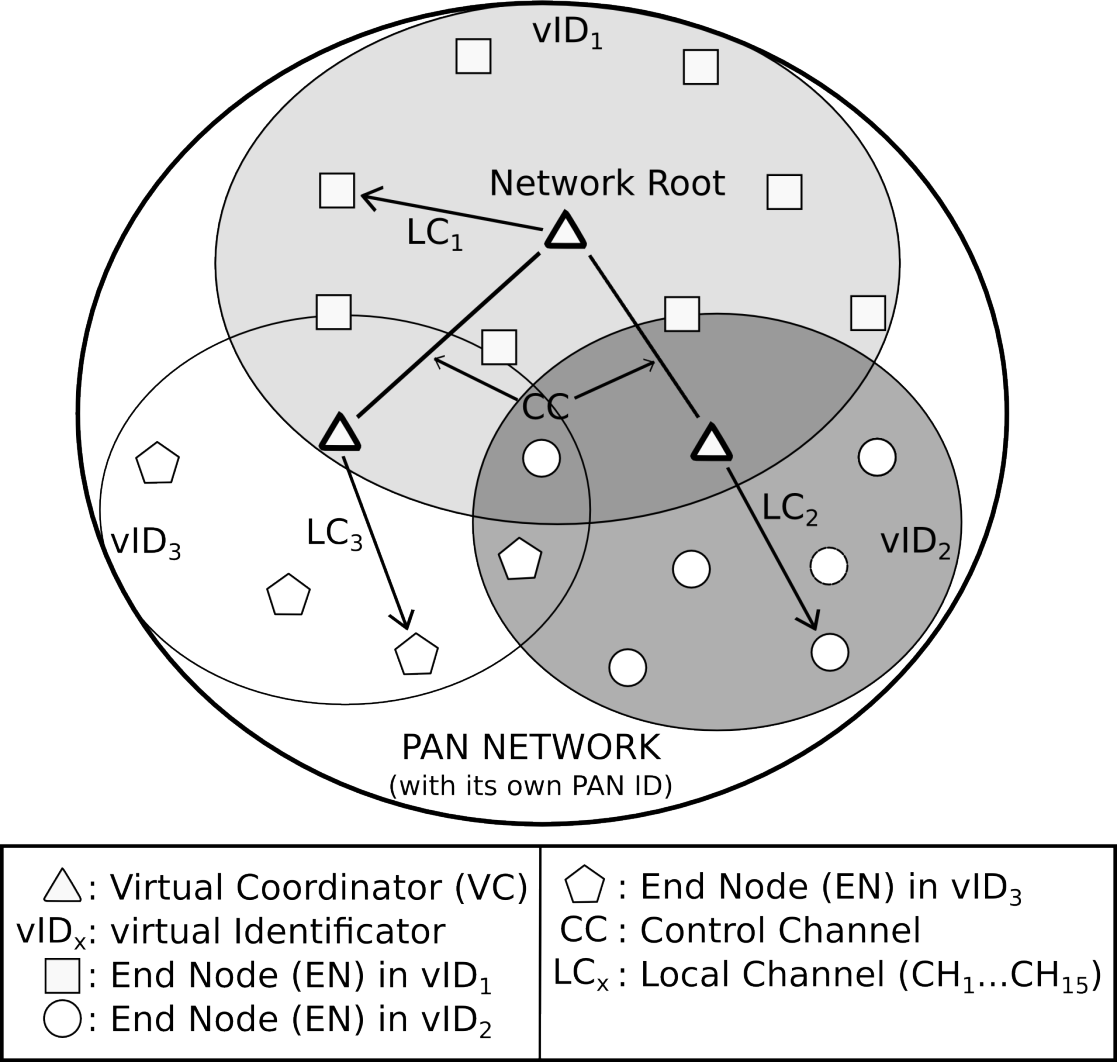
*mDARAL* basic concepts.

The virtual sub-network concept developed in *DARAL* is exploited to support a pre-defined control channel. As long as the devices use a multi-radio approach (considering multi-radio as the use of two radios), one of the radios is assigned to this control channel and the other one to the communication within the sub-network, as it is shown in Figure 1. This behaviour involves only virtual coordinators (VCs) being suitable to use both radios regularly. For end nodes (ENs), the second radio is usually switched off and it would be switched on only for some specific operations like channel migration or channel analysis. This modification to be able to use different radios leads to changes in several parts of *DARAL* and *DRSP*, which are explained in detail as follows.

### 3.1. Cluster Creation Using the DRSP Algorithm in a Multi-Radio Environment

**Figure 2:**
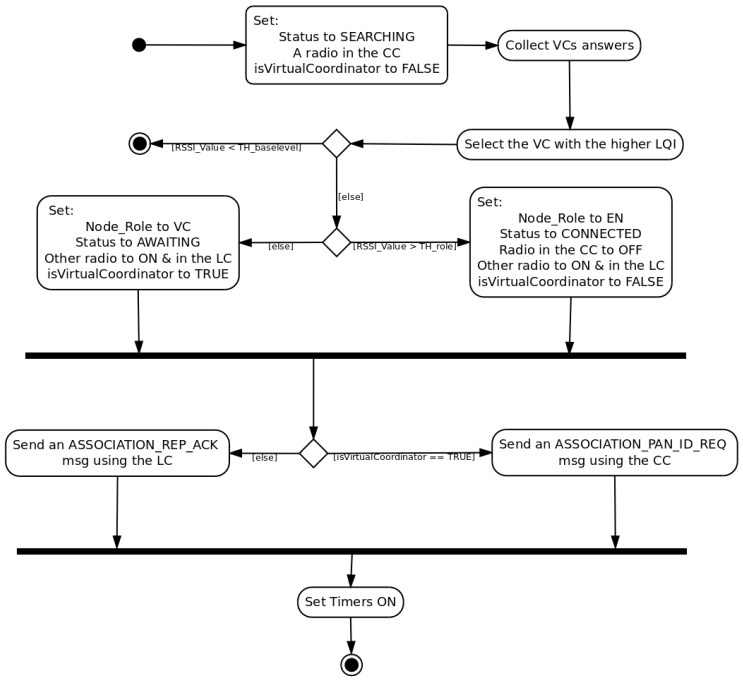
Dynamical Role Selection Process (DRSP) with multi-channel use.

Despite the fact that the role selection is still based on the link quality indicator between a VC and an EN, *mDARAL* leads to several minor changes in this process. When an EN starts to send an Association Request message waiting for an answer of a VC, it uses the control channel (CC) as it is set as the standard pre-defined communication channel. VCs include, in the *Association Reply* message, information about the secondary channel or local channel (LC) use within its sub-network, being the information stored for the selection process. Consequently, once a node has selected a VC as a parent, it turns off its primary radio and sends the Association Reply Acknowledgement through the secondary radio using the local channel information received during the association polling process. Figure 2 shows a graphical UML (Unified Modeling Language) description of the algorithm, including the radio status changes.

**Figure 3:**
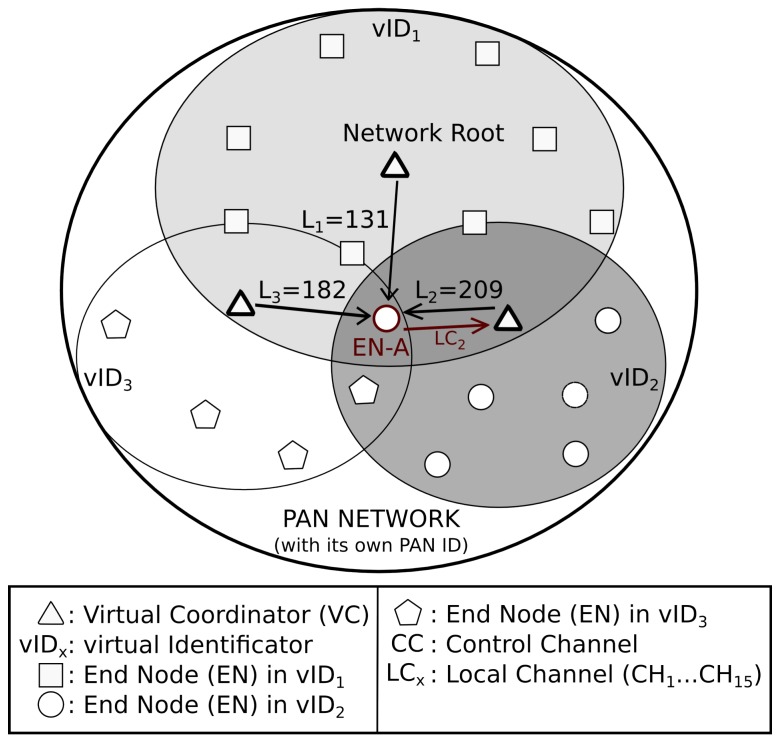
Example of link selection and role adoption process.

Figure 3 shows a particular example, where node *EN-A* connects to node $VC_{2}$, answering through channel $LC_{2}$. It is important to mention that every Association Reply contains the information about the LC for that VC, thus the node running *DRSP* stores this information for all the gathered answers while it is trying to get connected to the network.

This strategy tries to reduce the power consumption of ENs, keeping only one radio active during most of the communication time.

### 3.2. Multi-Radio Management in mDARAL

This section details the changes in *DARAL* in order to achieve a multi-radio version. First of all, each VC creates its sub-network based on a random channel selection, excluding from this random selection the control channel which is isolated from the rest of the available channels for creating virtual sub-networks.

*mDARAL* has been designed to change the LC under two different scenarios. The former is associated with a timer specifically configured and triggered when the communication link has been lost. The latter is used when degradation in the link-quality is detected. The estimation of the link-quality is not a trivial task in these kinds of networks. The existing solutions applicable to wireless networks are not directly usable in WSNs [28]. Link-quality metrics can be classified in physical, such as Received Signal Strength Indication (RSSI), Signal-to-Noise Ratio (SNR), or LQI, and logical metrics (Packet Success Rate, Require Number of Packets, Expected Transmission Count, among others). The use of physical metrics has several advantages: (i) there are no additional costs because its measurement is directly done by the hardware of the radio; (ii) it is possible to obtain a measurement with a small number of packets for approximating the quality of the link; and (iii) there is no need for broadcasting packets in order to measure the link. Taking into account these reasons, we decided to develope *mDARAL* using physical metrics.

Srinivasan et al. [29] performed a study for comparing RSSI and LQI as link quality metrics. In this study, they concluded that RSSI is a promising indicator for measuring isolated packets. However, the mean LQI computed over many packets has a better correlation with Packet Delivery Ratio (PDR). In this work, we have decided to use the LQI parameter for getting an average value of the link quality along the network lifetime. For detecting when the LQI falls below a threshold, we use Equation (1). This equation uses the LQI measured during the connection phase, setting it as initial average LQI ($avgLQI_{X}$). Using this parameter jointly with a user-defined tolerance *rssiPctLst*, it is possible to set the minimum LQI level up to the channel migration that should be fired

(1) $$minQoS=avgLQI_{X}\times rssiPctLst.$$

**Figure 4:**
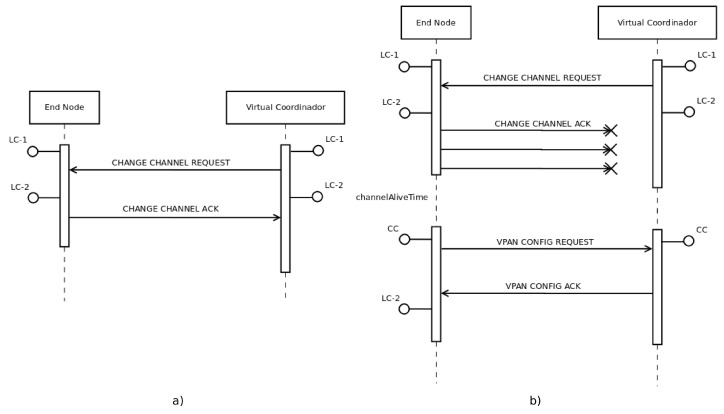
Message flow for channel migration. (**a**) normal flow; (**b**) flow with errors.

The selection of a new channel has been randomized for this version of *mDARAL*, excluding the control channel and the current channel from the potential channels. Figure 4 shows how the channel migration process is carried out using two new messages, *Channel Change Request*, sent by VCs, and *Channel Change ACK*, sent by ENs (see Appendix A for further information). A VC leads the channel migration, which will only be triggered if a fixed percentage of ENs in the sub-network falls below the *minQoS* value. This percentage is based on a new parameter, $ratSnsngAckRd_{X}$, which is used in Equation (2), where $numAnswers_{X}$ is the number of nodes that have answered the request in a certain sub-network *X*, and ($numNodes_{X}$) is the total number of ENs in that sub-network.

After starting the channel migration for the LC, the VC should receive the different ACKs from every node in the sub-network, confirming its connection in the new channel

(2) $$ratSnsngAckRd_{X}=\frac{numAnswers_{X}}{numNodes_{X}}.$$

Finally, it is necessary to consider the possibility of a connectivity problem due to the channel migration. Therefore, a node, which is connected to a certain sub-network, could request the sub-network configuration anytime, using the CC and the messages *VPAN Config Request* and *VPAN Config ACK* (see the Appendix A for further information). In both cases, the VPAN (Virtual Private Ad hoc Network) concept is related to the sub-network created, and it is identified as vID (virtual identification).

## 4. Simulation Testbed

The present section describes the simulation environment used for the performance analysis of *mDARAL*, considering different scenarios and conditions. In order to simulate *mDARAL*, a widely used simulator like *OMNeT++* [30] has been used. *OMNeT++* is an event simulator, which is not specifically focused on WSN. Therefore, the well-known communication framework inetmanet [31] has been used. In order to use inetmanet properly, it has been necessary to modify the physical layer, notification board and battery modules, allowing the use of a second radio in the same module. These changes allow the detection of the radio status (sending, receiving, sleeping, disconnected) for each module. The changes do not provide any special functionality, but they are necessary to operate the simulation model successfully. It is also necessary to remark that *OMNeT++* does not provide an efficient utility to simulate multi-channel energy detection and the cost of doing it manually dramatically impacts the performance of the simulator. Due to this issue, this work has fired the channel migration through a timer, and it has selected the channel to migrate randomly from a set of channels, which has covered the 15 channels of *IEEE 802.15.4*, excluding the CC and the current LC. *mDARAL* is publicly available in [24].

Based on [1], a Smart City scenario proposal has been developed for the simulations carried out in the present work. The nodes for this Smart City scenario have been randomly distributed. The communication band chosen for these experiments has been the ISM 2.4 GHz from the *IEEE802154RadioModel* of inetmanet, using its beaconless mode. Due to the multi-radio nature of this study and to the single-radio model implementation available in inetmanet and *OMNeT++*, it has been necessary to create a new multi-radio version of the radio and the *Medium Access Control (MAC)* sub-modules under the NIC (Network Interface Card) module.

**Figure 5:**
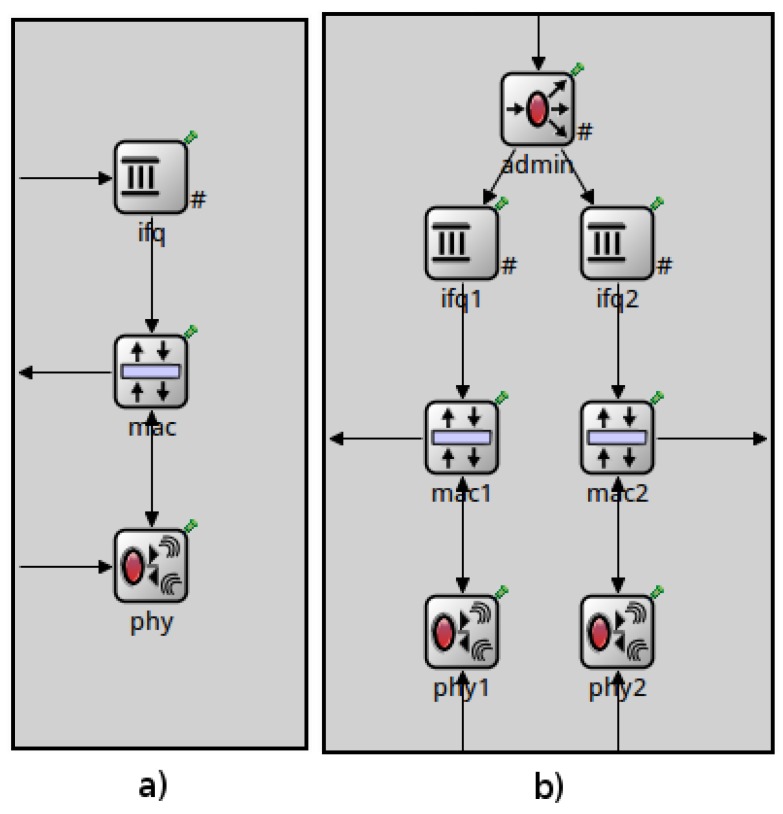
(**a**) single-radio NIC module; (**b**) multi-radio NIC module.

- NIC Module: This module contains all the layers related to the *IEEE 802.15.4*, MAC and PHY (Physical) layers, and a queue module, which manages the packets received from upper layers. Now, the NIC module contains two of each sub-modules and an additional sub-module to evaluate and assign the packets from the upper layer to a certain radio, as it is shown in Figure 5b.
- MAC Module: In order to allow tasks like channel change, connection and disconnection required by the new protocol, the MAC module has been modified to receive requests from upper layers. This change allows the sending of *IEEE 802.15.4* primitives to change the channel or the radio state. Thus, as the current MAC module in the inetmanet does not include all the *IEEE 802.15.4* primitives, it has been necessary to include them.

Additionally to the changes in these modules, some noise generator nodes have been added. These nodes generate noise in the form of messages in order to increase the traffic in the channels. The nodes are not recognised by any VC in the network, so they are not connected to the network focus of our study. From these nodes, 50% are fixed in their positions and the other 50% are mobile nodes, which change their positions every 30 s at a fixed speed of 1 m/s.

Several experiments were carried out using the recommended configuration suggested in [5]. For simulation purposes, a timer (*channelAliveTime*) has been used, which defines the time channel migration for a sub-network, firing the channel migration process, and a parameter defining the number of nodes generating noise (numNoisyNodes), as a percentage of the number of total nodes present in the network. All the parameters related to the simulations are shown in Table 1 jointly with their values. Appendix A describes in detail the new parameters and messages of *mDARAL*.

Smart City scenarios present different traffic conditions, which makes it necessary to define different traffic workloads for the present work. As it was carried out in our previous work, the node density (ND) is also considered for the present simulations, using 5, 10 and 15. These different ND factors result in the use of different area sizes such as 145 × 145 m or 700 × 700 m area. The traffic is not only defined by the ND, but the time interval (TI) between sending is also a critical factor in terms of traffic analysis. For this purpose, two statistically independent values have been considered: 1 and 30 s. Finally, it is necessary to mention that this work considers two different propagation models (PM) in order to develop a fair comparison. As it is shown in [26], the use of a deterministic and a probabilistic propagation model is recommended, so, for this work, the *Free Space Model* (FS) is selected as a deterministic model and the *Log-Normal Shadowing* (LNS) as a probabilistic model, resulting in a large comparison. The different scenarios simulated are shown in Table 2. Additionally, every scenario has been simulated 10 times, using different randomly generated scenarios.

### 4.1. Metrics Overview

This section presents the different metrics used for the present research, describing the parameters that have been measured.

#### 4.1.1. Convergence Time

One of the main parameters studied in the present work is the convergence time, which has been calculated as the elapsed time since the node has been switched on until the node changes its internal state machine to *CONNECTED* or *AWAITING*. Finally, the convergence time is presented as the average of all the convergence times in the network.

#### 4.1.2. Number of Control Messages Sent during the Set-Up Phase

Another one of the main parameters in this work is the number of control messages sent during the set-up phase, which is highly dependent on the number of control messages sent by each node. The control messages taken into account during the set-up phase are the *ASSOCIATION_REQ* and *ASSOCIATION_REP*. The result in terms of number of messages is obtained as the mean number of messages sent by all of the nodes.

#### 4.1.3. Energy Consumption during the Set-Up Phase

The energy consumption has been measured during the set-up phase in order to measure the impact of multi-radio approaches during the network forming phase. It is necessary to point out the importance of intelligent energy management methods in cooperative transmissions such as the multi-radio systems presented in [32] by Sheng et al. They demonstrated that, through the use of power-allocation methods, it is possible to reduce the total power consumption while maintaining the required QoS, which is highly necessary for these systems. For this study, the processing time for the channel selection has been excluded, focusing exclusively on connection time of the network, regardless of the multi-radio configuration.

The different configuration parameters related to energy, such as battery capacity and consumption configuration, have been identically defined, obtaining directly comparable results in *mWs*. The energy consumption has been measured through a variable that has stored the remaining energy in its battery, allowing for calculating the energy used during the experiment. The result is presented as the mean of the energy consumption for all nodes in the net.

## 5. Results

The following section is focused on the performance analysis of *mDARAL* compared to *DARAL* under the simulation scenarios showed in Table 2. In these scenarios, several parameters, as in previous works [5], were taken into account. Thus, convergence time, number of messages and consumed battery during set-up time are the parameters used for comparison purposes between the mentioned protocols.

**Figure 6:**
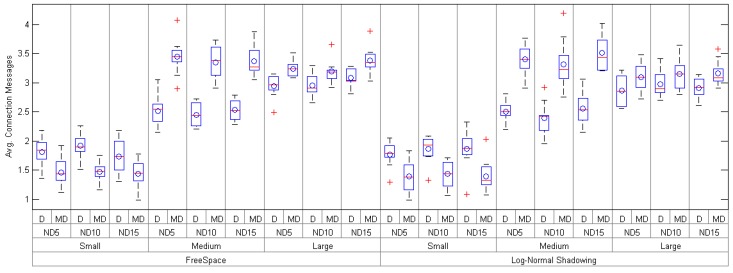
Average number of control messages of *Daral* (D) and *mDaral* (MD) for small-, medium- and large-size scenarios under different propagation conditions and node densities (ND5, ND10 and ND15).

Attending to the obtained results in terms of number of messages (see Table 3 and Figure 6), *mDARAL* gets a larger amount of them except in small networks, during network set-up compared to *DARAL*, which is completely reasonable taking into account how *mDARAL* manages the connection messages. Analyzing the scenario formed by a large number of nodes with a node density of 15, it is noticed that *DARAL* needs 3.09 messages per node during the set-up compared to the 3.38 messages used by *mDARAL* for the Freespace propagation configuration. On the contrary, when the Log-Normal Shadowing configuration is used, both approaches reduce the average number of messages during the set-up with 3.91 and 2.91 for *mDARAL* and *DARAL*, respectively. Despite the number of messages being larger for *mDARAL*, during network set-up, the energy consumption is not penalized by this circumstance, as *mDARAL* optimizes the use of batteries under all testbed scenarios, especially when a Log-Normal Shadowing with a large number of nodes and node density equal to 15 is deployed.

**Figure 7:**
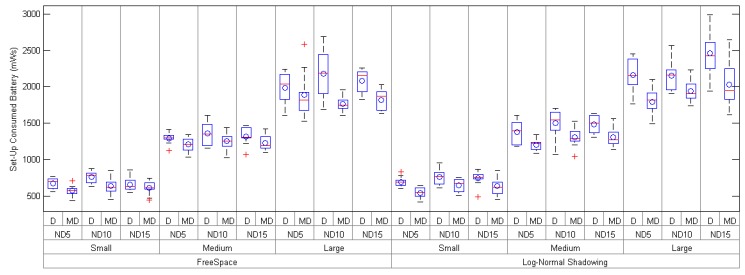
Average battery consumed during the network set-up of *Daral* (D) and *mDaral* (MD) for small-, medium- and large-size scenarios under different propagation conditions and node densities (ND5, ND10 and ND15).

The explanation of why *mDARAL* has a better energy consumption (see Table 4 and Figure 7) has to be focused while attending to how the radios are managed. As it was explained previously, *mDARAL* maintains one of the radios switched off as much time as possible, and, for the set-up time, the second radio is only used in order to acknowledge the connection of ENs through the LC, resulting in a minimum time usage of the second radio, considering that the first radio switches off at the same time that the second is switched on. Probably, the use of LCs, instead of the CC for the whole communication in the network, reduces the number of collisions, increasing the rate of packets successfully delivered. Therefore, the convergence time is also reduced, even though the number of control messages sent is increased.

**Figure 8:**
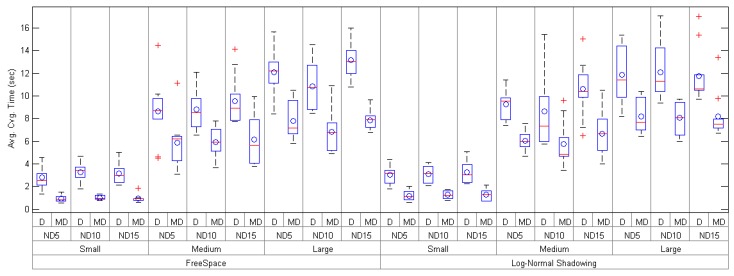
Average convergence time of *Daral* (D) and *mDaral* (MD) for small-, medium- and large-size scenarios under different propagation conditions and node densities (ND5, ND10 and ND15).

Table 5 and Figure 8 show the behaviour of the convergence time for both routing algorithms, and also for the FreeSpace and the Log-Normal Shadowing configurations. In general terms, it can be seen how *mDARAL* reduces the time for creating the entire network under all the possible analyzed scenarios. For the FreeSpace configuration with a node density of 15, *mDARAL* gets an average improvement between 3.35× (Small) and 1.67× (Large), while, in the Log-Normal Shadowing configuration, the values are in a range from 2.56× (Small) to 1.43× (Large).

Finally, as our previous works show, it would be convenient to calibrate the parameters of the algorithm in terms of energy consumption reduction. The convergence time has shown a higher impact on the energy consumption than the number of messages sent.

## 6. Conclusions

This paper presents an evolution of the *DARAL* routing algorithm focused on multi-radio environments like Smart Cities. We analyzed three key parameters for WSNs during the set-up phase: (i) the convergence time; (ii) the number of control messages (control overhead); and (iii) the energy consumption. We have carried out intense experiments intending to validate the multi-radio routing algorithm presented in this work. In order to make a fair analysis, a comparison with the single-radio protocol has been developed, considering different environmental factors within the process.

After the analysis of the results, it has been found that *mDARAL* presents a lower number of control messages during the set-up phase for small scenarios under different node densities and propagation models. For medium and large scenarios, the presented configuration of the algorithms results in a better performance of *DARAL* under different node densities and propagation conditions. It is necessary to point out that the differences between *DARAL* and *mDARAL* in terms of number of control messages sent are small enough to consider the results similar. The consistency of the results measured is very similar, going from 0.12 to 0.31 and it does not present any relevant difference. Taking into account the convergence time, it is noticed that *mDARAL* decreases the convergence time in all of the considered scenarios, even for different propagation conditions and densities. Moreover, *mDARAL* shows better results and consistency in terms of stability.

*mDARAL* shows a better energy consumption compared to *DARAL* under all the scenarios tested in this work. In our analysis, the propagation model seems to impact the consumption, resulting in the LogNormal Shadowing configuration having a larger difference between models. Finally, *mDARAL* presents an overall improvement in comparison with *DARAL*.

## Figures and Tables

**Table 1:** Main configuration parameters for the simulation.

Parameter	Value
Carrier Frequency	2.4 GHz
Carrier Sense Sensitivity	−85 dBm
Transmit Power	1.0 mW
$T_{link}$	10.0 s
$T_{alive}$	600.0 s
$T_{down}$	5.0 s
$T_{reconnect}$	2.0 s
$T_{ack}$	1.5 s
$L_{nodes}$	50
$TH_{baselevel}$	45
$TH_{role}$	130
*rssiPctLst*	0.3
*ratSnsngAckRd*	0.3
*channelAliveTime*	250.0 s
*numChannels*	10
Payload Size	70 Bytes
$MAX_{SimTime}$	3600 s

**Table 2:** Simulation scenarios for *mDaral*. ND, TI and PM stand for Node Density, Time Interval and Propagation Model respectively.

Network Size	Number of Nodes	Area Size (m)	ND	TI (s)	PM
Small	100	250 × 250	5	1	FS
Small	100	250 × 250	5	1	LNS
Small	100	250 × 250	5	30	FS
Small	100	250 × 250	5	30	LNS
Small	100	175 × 175	10	1	FS
Small	100	175 × 175	10	1	LNS
Small	100	175 × 175	10	30	FS
Small	100	175 × 175	10	30	LNS
Small	100	145 × 145	15	1	FS
Small	100	145 × 145	15	1	LNS
Small	100	145 × 145	15	30	FS
Small	100	145 × 145	15	30	LNS
Medium	400	500 × 500	5	1	FS
Medium	400	500 × 500	5	1	LNS
Medium	400	500 × 500	5	30	FS
Medium	400	500 × 500	5	30	LNS
Medium	400	350 × 350	10	1	FS
Medium	400	350 × 350	10	1	LNS
Medium	400	350 × 350	10	30	FS
Medium	400	350 × 350	10	30	LNS
Medium	400	290 × 290	15	1	FS
Medium	400	290 × 290	15	1	LNS
Medium	400	290 × 290	15	30	FS
Medium	400	290 × 290	15	30	LNS
Large	800	700 × 700	5	1	FS
Large	800	700 × 700	5	1	LNS
Large	800	700 × 700	5	30	FS
Large	800	700 × 700	5	30	LNS
Large	800	500 × 500	10	1	FS
Large	800	500 × 500	10	1	LNS
Large	800	500 × 500	10	30	FS
Large	800	500 × 500	10	30	LNS
Large	800	400 × 400	15	1	FS
Large	800	400 × 400	15	1	LNS
Large	800	400 × 400	15	30	FS
Large	800	400 × 400	15	30	LNS
FS: Free Space Model		LNS: Log-Normal Shadowing			

**Table 3:** Average number of control messages for small-, medium- and large-size scenarios under different propagation conditions. ND and PM stand for Node Density and Propagation Model respectively.

PM	ND	Algorithm	Avg ± Dev Small-	Avg ± Dev Medium-	Avg ± Dev Large-Scenario
FS	5	*DARAL*	1.81 ± 0.26	1.92 ± 0.23	1.74 ± 0.29
FS	5	*mDARAL*	1.46 ± 0.25	1.47 ± 0.20	1.44 ± 0.22
FS	10	*DARAL*	2.51 ± 0.26	2.45 ± 0.20	2.54 ± 0.19
FS	10	*mDARAL*	3.44 ± 0.31	3.35 ± 0.27	3.37 ± 0.27
FS	15	*DARAL*	2.94 ± 0.2	2.96 ± 0.19	3.09 ± 0.14
FS	15	*mDARAL*	3.24 ± 0.12	3.19 ± 0.19	3.38 ± 0.23
LNS	5	*DARAL*	1.77 ± 0.21	1.87 ± 0.23	1.86 ± 0.33
LNS	5	*mDARAL*	1.4 ± 0.28	1.44 ± 0.21	1.39 ± 0.28
LNS	10	*DARAL*	2.51 ± 0.17	2.4 ± 0.27	2.56 ± 0.28
LNS	10	*mDARAL*	3.4 ± 0.26	3.32 ± 0.42	3.51 ± 0.29
LNS	15	*DARAL*	2.87 ± 0.25	2.97 ± 0.22	2.91 ± 0.17
LNS	15	*mDARAL*	3.09 ± 0.25	3.15 ± 0.26	3.16 ± 0.21
		FS: Free Space Model		LNS: Log-Normal Shadowing	

**Table 4:** Average battery consumed during the network set-up for small-, medium- and large-size scenarios under different propagation conditions. ND and PM stand for Node Density and Propagation Model respectively.

PM	ND	Algorithm	Avg ± Dev Small-	Avg ± Dev Medium-	Avg ± Dev Large-Scenario
FS	5	*DARAL*	672.96 ± 79.57	763.34 ± 84.91	661.26 ± 94.36
FS	5	*mDARAL*	574.87 ± 72.71	636.53 ± 120.27	614.51 ± 95.19
FS	10	*DARAL*	1292.5 ± 77.82	1358.6 ± 159.1	1321.0 ± 118.45
FS	10	*mDARAL*	1208.6 ± 93.44	1258.3 ± 122.65	1227.1 ± 100.27
FS	15	*DARAL*	1990.5 ± 232.65	2183.9 ± 330.03	2084.7 ± 155.53
FS	15	*mDARAL*	1893.7 ± 314.9	1763.9 ± 105.63	1823.3 ± 139.14
LNS	5	*DARAL*	695.29 ± 70.03	766.69 ± 103.34	742.48 ± 103.03
LNS	5	*mDARAL*	544.92 ± 81.41	645.17 ± 96.48	635.82 ± 129.86
LNS	10	*DARAL*	1380.0 ± 153.53	1504.6 ± 192.83	1486.6 ± 127.98
LNS	10	*mDARAL*	1201.0 ± 81.34	1312.9 ± 141.33	1305.5 ± 125.45
LNS	15	*DARAL*	2165.8 ± 220.82	2152.9 ± 202.68	2457.3 ± 326.75
LNS	15	*mDARAL*	1794.9 ± 179.81	1941.1 ± 149.93	2032.9 ± 316.07
		FS: Free Space Model		LNS: Log-Normal Shadowing	

**Table 5:** Average convergence time for small-, medium- and large-size scenarios under different propagation conditions. ND and PM stand for Node Density and Propagation Model respectively.

PM	ND	Algorithm	Avg ± Dev Small-	Avg ± Dev Medium-	Avg ± Dev Large-Scenario
FS	5	*DARAL*	2.78 ± 1.03	3.24 ± 0.84	3.15 ± 0.91
FS	5	*mDARAL*	0.94 ± 0.32	1.01 ± 0.2	0.94 ± 0.36
FS	10	*DARAL*	8.62 ± 2.82	8.83 ± 1.66	9.55 ± 2.21
FS	10	*mDARAL*	5.89 ± 2.26	5.91 ± 1.35	6.13 ± 2.17
FS	15	*DARAL*	12.09 ± 1.89	10.84 ± 2.10	13.15 ± 1.48
FS	15	*mDARAL*	7.76 ± 1.59	6.85 ± 1.81	7.86 ± 0.84
LNS	5	*DARAL*	3.06 ± 0.82	3.11 ± 0.75	3.28 ± 0.96
LNS	5	*mDARAL*	1.19 ± 0.46	1.28 ± 0.36	1.28 ± 0.49
LNS	10	*DARAL*	9.26±1.3	8.62±3.18	10.6±2.5
LNS	10	*mDARAL*	6.01 ± 0.89	5.77 ± 1.99	6.63 ± 2.03
LNS	15	*DARAL*	11.87 ± 2.6	12.07 ± 2.45	11.73 ± 2.44
LNS	15	*mDARAL*	8.21 ± 1.51	8.07 ± 1.43	8.19 ± 2.02
		FS: Free Space Model		LNS: Log-Normal Shadowing	

## Appendix A. mDARAL Changes

This appendix lists the changes in *mDARAL* with respect to *DARAL*, covering the additional control messages in the new version of the routing algorithm and the new parameters included to configure the new features. This appendix is based on Appendixes A and B of our previous work [5].

Operation code represents the control message identification used by *DARAL* and *mDARAL*, which counts 19 different messages, 15 from *DARAL* and an additional four from *mDARAL*, which are described below:

- CHANGE_CHANNEL_REQUEST: it fires the channel migration process.
- CHANGE_CHANNEL_ACK: it confirms the reception of a *CHANGE_CHANNEL_REQUEST* frame.
- VPAN_CONFIG_REQUEST: it sends a request to the VC of a certain sub-network, requesting the configuration information of its sub-network (LC and vIDs).
- VPAN_CONFIG_ACK: it confirms the reception of a *VPAN_CONFIG_REQUEST* frame, broadcasting the sub-network configuration details through the CC.

The following list presents the additional parameters of *mDARAL*, with respect to *DARAL*:

- *rrsiPctLst*: This parameter defines the maximum percentage of link quality, in terms of LQI/RSSII, that the link can worsen. In addition, 100% of the signal is set by the initial LQI measurement used during the connection with the VC.
- *ratSnsngAckRd*: This ratio sets the limit where the VC can start with the migration channel process. After receiving enough answers to the *CHANGE_CHANNEL_REQUEST* from the nodes in its sub-network, the VC changes its LC.
- *channelAliveTime*: This timer defines the time that a node can wait without any answer once the migration channel process has been fired. After this time, the node requests the configuration of the sub-network to the VC, using a *VPAN_CONFIG_REQUEST* through the CC.

All of these parameters could be tuned, as in *DARAL*, by intelligent systems, making feasible their application to diverse scenarios, not only for Smart Cities, but also for IoT.

## References

[1] Sanchez L., Munoz L., Galache J.A., Sotres P., Santana J.R., Gutierrez V., Ramdhany R., Gluhak A., Krco S., Theodoridis E. (2014). SmartSantander: IoT experimentation over a smart city testbed. Comput. Netw..

[2] Rong W., Xiong Z., Cooper D., Li C., Sheng H. (2014). Smart City Architecture: A technology guide for implementation and design challenges. China Commun..

[3] RaspBerry Pi Zero. https://www.raspberrypi.org/blog/raspberry-pi-zero/.

[4] Estevez F.-J., Rebel G., González J., Gloesekoetter P. (2014). DARP: Dynamic and adaptive radio protocol for Wireless Sensor Networks. Electron. Lett..

[5] Estevez F.J., Glosekotter P., Gonzalez J. (2016). DARAL: A Dynamic and Adaptive Routing Algorithm for Wireless Sensor Networks. Sensors.

[6] IEEE Std. 802.15.1 IEEE Standard for Information Technology—Telecommunications and Information Exchange between Systems—Local and Metropolitan Area Networks—Specific Requirements. Part 15.1: Wireless Medium Access Control (MAC) and Physical Layer (PHY) Specifications for Wireless Personal Area Networks (WPANs). http://standards.ieee.org/getieee802/download/802.15.1-2005.pdf.

[7] IEEE Std. 802.15.3 IEEE Standard for Information Technology—Telecommunications and Information Exchange between Systems—Local and Metropolitan Area Networks—Specific Requirements. Part 15.3: Wireless Medium Access Control (MAC) and Physical Layer (PHY) Specifications for High Rate Wireless Personal Area Networks (WPANs) Amendment 2: Millimeter-wave-based Alternative Physical Layer Extension. http://standards.ieee.org/getieee802/download/802.15.3c-2009.pdf.

[8] IEEE 802.15.4 IEEE Standard for Local and Metropolitan Area Networks–Part 15.4: Low-Rate Wireless Personal Area Networks (LR-WPANs). http://standards.ieee.org/getieee802/download/802.15.4-2011.pdf.

[9] IEEE 802.15.5 IEEE Standard for Recommended Practice for Information Technology—Telecommunications and Information Exchange between Systems—Local and Metropolitan Area Networks—Specific Requirements Part 15.5: Mesh Topology Capability in Wireless Personal Area Networks (WPANs). http://standards.ieee.org/getieee802/download/802.15.5-2009.pdf.

[10] Winter T., Thubert P., Brandt A., Hui J., Kelsey R., Levis P., Pister K., Struik R., Vasseur J.P., Alexander R. RPL: IPv6 Routing Protocol for Low Power and Lossy Networks. https://tools.ietf.org/html/rfc6550.

[11] Perkins C., Belding-Royer E., Das S. Ad Hoc On-Demand Distance Vector (AODV) Routing. https://www.ietf.org/rfc/rfc3561.

[12] Mogaibel H.A., Othman M., Subramaniam S., Hamid N. (2016). Review of channel assignment approaches in multi-radio multi-channel wireless mesh network. J. Netw. Comput. Appl..

[13] Bahl P., Adya A., Padhye J., Wolman A. (2004). Reconsidering Wireless Systems with Multiple Radios. SIGCOMM Comput. Commun. Rev..

[14] Kusy B., Richter C., Hu W., Afanasyev M., Jurdak R., Brunig M. Radio diversity for reliable communication in WSNs. Proceedings of the 10th International Conference on Information Processing in Sensor Networks (IPSN).

[15] Liu C.H., Gkelias A., Hou Y., Leung K.K. (2009). Cross-layer design for QoS in wireless mesh networks. Wirel. Pers. Commun..

[16] Saravanan M., Madheswaran M. (2014). A Hybrid Optimized Weighted Minimum Spanning Tree for the Shortest Intrapath Selection in Wireless Sensor Network. Math. Probl. Eng..

[17] Wang D.J. (2010). Clustering mesh-like wireless sensor networks with an energy-efficient scheme. Int. J. Sens. Netw..

[18] Roul R.R., Ghosh S.K. (2014). Adaptive data aggregation and energy efficiency using network coding in a clustered wireless sensor network: An analytical approach. Comput. Commun..

[19] Jian Z., Jun L., Zhao H., Bi Y.G. (2016). Research on routing protocol facing to signal conflicting in link quality guaranteed WSN. Wirel. Netw..

[20] Diallo C., Marot M., Becker M. Link Quality and Local Load Balancing Routing Mechanisms in Wireless Sensor Networks. Proceedings of the Sixth Advanced International Conference on Telecommunications (AICT).

[21] Machado K., Rosario D., Cerqueira E., Loureiro A.A.F., Neto A., Neumann de Souza J. (2013). A Routing Protocol Based on Energy and Link Quality for Internet of Things Applications. Sensors.

[22] Gkelias A., Boccardi F., Liu C.H., Leung K.K. MIMO routing with QoS provisioning. Proceedings of the 3rd International Symposium on Wireless Pervasive Computing.

[23] Estevez F.J. (2016). DARP: A New Routing Algorithm for Large Communication Infrastructures. Ph.D. Thesis.

[24] Estevez F.J., García J.M. DARAL Simulation Code for OMNeT++. https://github.com/fjestevez/MDARP.

[25] Lee I.G., Kim M. (2016). Interference-aware self-optimizing Wi-Fi for high efficiency internet of things in dense networks. Comput. Commun..

[26] Estevez F.J., Garcia J.M., Castillo-Secilla J.M., Gonzalez J., Gloesekoetter P. (2016). Enabling Validation of IEEE 802.15.4 Performance through a New Dual-Radio Omnet plus plus Model. Elektron. Electrotech..

[27] Joshi G.P., Kim S.W. (2016). A Survey on Node Clustering in Cognitive Radio Wireless Sensor Networks. Sensors.

[28] Renner C., Ernst S., Weyer C., Turau T. Prediction accuracy of link-quality estimators. Proceedings of the 8th European Conference on Wireless Sensor Networks.

[29] Srinivasan K., Levis P. RSSI is Under Appreciated. Proceedings of the Third Workshop on Embedded Networked Sensors (EmNets).

[30] Varga A. The omnet++ discrete event simulation systems. Proceedings of the European Simulation Multiconference.

[31] Inetmanet Framework for Wireless Sensor and Ad-Hoc Networks Using OMNeT++. https://github.com/aarizaq/inetmanet-2.0.

[32] Sheng Z., Fan J., Liu C.H., Leung V.C., Liu X., Leung K.K. (2015). Energy-efficient relay selection for cooperative relaying in wireless multimedia networks. IEEE Trans. Veh. Technol..

